# Influence of diabetes on mortality and ICD therapies in ICD recipients: a systematic review and meta-analysis of 162,780 patients

**DOI:** 10.1186/s12933-022-01580-y

**Published:** 2022-07-29

**Authors:** Hualong Liu, Jinzhu Hu, Wen Zhuo, Rong Wan, Kui Hong

**Affiliations:** 1 Department of Cardiovascular Medicine, The Second Affiliated Hospital of Nanchang University, No. 1, Minde Road, Nanchang, 330006 Jiangxi China; 2 Jiangxi Key Laboratory of Molecular Medicine, The Second Affiliated Hospital of Nanchang University, Nanchang, Jiangxi China

**Keywords:** Diabetes, Influence, Mortality, ICD therapies, ICD recipients

## Abstract

**Background:**

The influence of diabetes on the mortality and risk of implantable cardioverter defibrillator (ICD) therapies is still controversial, and a comprehensive assessment is lacking. We performed this systematic review and meta-analysis to address this controversy.

**Methods:**

We systematically searched the PubMed, Embase, Web of Science and Cochrane Library databases to collect relevant literature. Fixed and random effects models were used to estimate the hazard ratio (HR) with 95% CIs.

**Results:**

Thirty-six articles reporting on 162,780 ICD recipients were included in this analysis. Compared with nondiabetic ICD recipients, diabetic ICD recipients had higher all-cause mortality (HR = 1.45, 95% CI 1.36–1.55). The subgroup analysis showed that secondary prevention patients with diabetes may suffer a higher risk of all-cause mortality (HR = 1.89, 95% CI 1.56–2.28) (for subgroup analysis, P = 0.03). Cardiac mortality was also higher in ICD recipients with diabetes (HR = 1.68, 95% CI 1.35–2.08). However, diabetes had no significant effect on the risks of ICD therapies, including appropriate or inappropriate therapy, appropriate or inappropriate shock and appropriate anti-tachycardia pacing (ATP). Diabetes was associated with a decreased risk of inappropriate ATP (HR = 0.56, 95% CI 0.39–0.79).

**Conclusion:**

Diabetes is associated with an increased risk of mortality in ICD recipients, especially in the secondary prevention patients, but does not significantly influence the risks of ICD therapies, indicating that the increased mortality of ICD recipients with diabetes may not be caused by arrhythmias. The survival benefits of ICD treatment in diabetes patients are limited.

**Supplementary Information:**

The online version contains supplementary material available at 10.1186/s12933-022-01580-y.

## Introduction

According to the latest data released by the International Diabetes Federation, the number of adult diabetic patients worldwide reached 537 million in 2021, and approximately 6.7 million people died of diabetes or diabetic complications, accounting for 12.2% of all-cause mortality [1]. Patients with diabetes have a higher risk of cardiovascular disease and mortality [2]. Heart failure (HF) is an end-stage clinical manifestation of organic heart disease and has become a major public health problem worldwide.

The prevalence of diabetes is 24% in chronic HF patients and up to 40% in hospitalized HF patients. Studies have shown that diabetes is an independent predictor of sudden cardiac death (SCD) in patients with HF and is associated with an increased risk of mortality [3, 4]. For example, in postinfarction patients, the mortality in the diabetic group was higher than that in the nondiabetic group [5]. It has been proven that implantable cardioverter defibrillator (ICD) can effectively prevent SCD and terminate malignant arrhythmias such as persistent ventricular tachycardia and ventricular fibrillation. Because of this unique property, ICD has been recommended as a class I recommendation to prevent SCD in patients with ischemic and nonischemic HF in current guideline [6]. Since diabetes generates a higher risk of SCD in HF patients, ICD implantation would be expected to have additional survival benefits.

To date, the influence of diabetes on the mortality and risk of ICD therapy is still controversial, and a comprehensive assessment is lacking. We performed this systematic review and meta-analysis to address this controversy.

## Methods

This article was prepared according to the Preferred Reporting Items for Systematic Reviews and Meta-Analyses (PRISMA) guidelines [7].

### Search strategy

The meta-analysis was conducted according to the PRISMA guidelines. Two authors (H.-L.L and W.Z.) systematically searched the PubMed, Embase and Cochrane Library from through February 28, 2022 for relevant articles published in English. The search strategy was as follows: [(Diabetes Mellitus) OR (Diabetes)] AND (“Defibrillators, Implantable” OR “Implantable Defibrillators” OR “Implantable Defibrillator” OR “Cardioverter-Defibrillators, Implantable” OR “Implantable Cardioverter-Defibrillator” OR “Implantable Cardioverter Defibrillators” OR “Defibrillator, Implantable”). Endnote X8 was used to manage the articles. The articles were independently selected by two authors (H.-L.L and J.-Z.H). After the title and abstract were reviewed and the off-topic articles were excluded, the full text of the remaining articles was screened against the inclusion criteria. Disagreements were resolved by discussion.

### Selection criteria

The studies were included if (1) the articles were published in English with available full texts; (2) the studies reported the mortality or risk of ICD therapy and (3) the studies provided the hazard ratio (HR), odds ratio (OR) or risk ratio (RR) as well as their corresponding 95% confidence intervals (CIs).

We excluded studies if (1) the articles were of certain types, such as reviews, meta-analyses, notes, and case reports; (2) the studies contained overlapping study populations or (3) the full text could not be found.

### Data extraction and quality assessment

Two reviewers (H.-L.L and W.Z.) independently extracted data from the included studies using a standard data extraction process. The following information was extracted from the articles: author’s name, publication year, study design, region of study, time frame, sample size, follow-up duration, age, sex ratio, region, time frame, left ventricular ejection fraction (LVEF), QRS duration, primary disease, prevention types, device implantation and outcomes.

The quality of the included studies was assessed independently by two reviewers (H.-L.L and J.-Z.H) using the Newcastle–Ottawa Scale (NOS). Each study was scored independently based on selection, comparability and outcome. We considered the article to be of high quality if it had a NOS score greater than 6. Disagreements were resolved by consensus.

### Outcomes and subgroups

The primary outcome was mortality in diabetic and nondiabetic ICD recipients, which was divided into all-cause mortality and cardiac mortality. A subgroup analysis of all-cause mortality was further performed by separating patients into ICD recipients for primary prevention, ICD recipients for secondary prevention and ICD recipients for primary or secondary prevention. The secondary outcome was the risk of ICD therapies in diabetic and nondiabetic ICD recipients, which was divided into appropriate therapy, inappropriate therapy, appropriate shock, inappropriate shock, appropriate anti-tachycardia pacing (ATP) and inappropriate ATP.

### Statistical analysis

Review Manager 5.3 (Cochrane Collaboration, Copenhagen, Denmark) was used to perform the meta-analysis. A sensitivity analysis was conducted to test the effect of individual studies using STATA version 12 (Stata Corporation, College Station, TX, USA). The natural logarithm of the hazard ratios (HRs) and its standard error (SElog HRs) were calculated. Heterogeneity was evaluated using chi-squared and I-squared tests. We considered there was substantial heterogeneity when I^2^ > 50%, and the random-effects model was used, otherwise, the fixed-effects model was used. Funnel plots as well as Begg and Egger test were drawn to evaluate the publication bias risk.

## Results

### Study selection and study characteristics

**Fig. 1:**
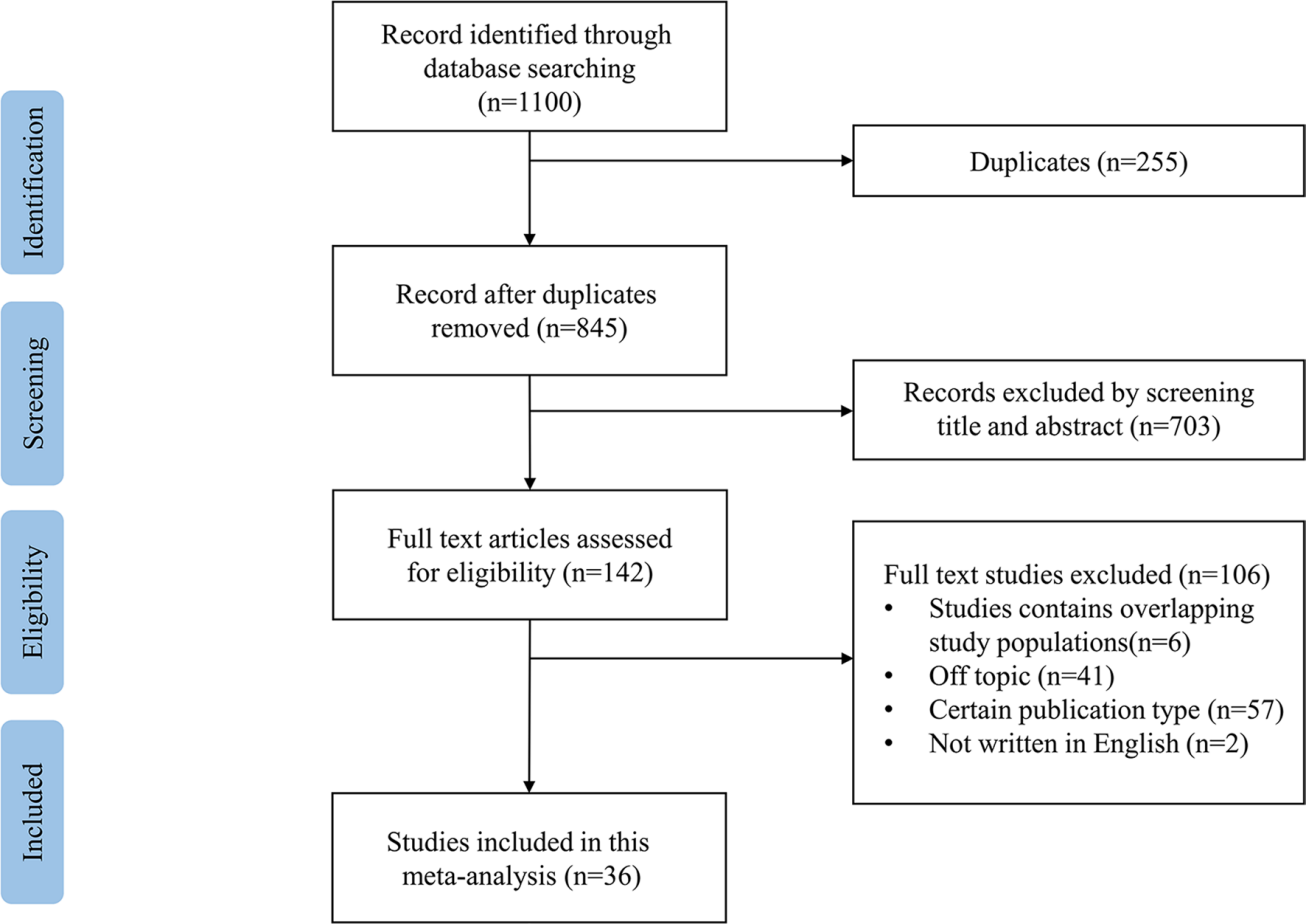
Flow diagram of the study selection process

We identified 1100 articles through electronic retrieval strategies. Of these, 255 were duplicates, and 703 were excluded because the articles did not meet the inclusion criteria. Of 142 articles screened for eligibility, 57 studies were unwanted publication types, 41 articles were off-topic, 6 studies had overlapping study populations, and 2 studies were not published in English. Finally, 36 studies [8–43] of 162,780 ICD recipients were included in the meta-analysis. The flow diagram of the literature inclusion process is shown in Fig. 1. Table 1 provides the main characteristics of the included studies, in addition to the regular index, including sample size, follow-up duration, region, time frame, age, sex ratio, LVEF, QRS duration, primary disease, device implantation, prevention types and outcomes. The quality of the included studies was assessed using the NOS, with an average NOS score of 7.55; the details of the quality assessment are shown in Table 2.

**Table 1:** Characteristic of included studies

Study	Study design	Region	Source	Time frame	Number of participants (N)	Age (year)	Male (%)	LVEF (%)	QRS (ms)	Follow-up duration (m)	Primary disease	Prevention types	Device implantation	Outcomes
Bilchick 2012	Retrospective study	USA	Centers for Medicare and Medicaid Services	2005–2007	45,884	72.5 (median)	76.0	NA	NA	Development cohort: 52.8 (50.4–55.2); validation cohort: 43.2 (37.2–48)^a^	HF	Primary	ICD	All-cause mortality
Borleffs 2009	Prospective study	Netherlands	Leiden University Medical Center	1996–2009	456	65.0 ± 10.0	86.0	35.0 ± 14.0	119.0 ± 30.0	54.0 ± 35.0	Ischaemic heart disease	Secondary	ICD	All-cause mortality
Briongos 2019	Prospective study	Spain	UMBRELLA	2006–2015	621	61.1 ± 11.4	87.3	26.6 ± 5.4	109.8 ± 25.3	52.8 ± 25.2	HF	Primary	ICD	All-cause mortality/cardiac mortality
Chao 2014	Retrospective study	Taiwan	Three Taiwan medical centers	1998–2009	238	63.0 ± 15.3	76.5	40.3 ± 13.3	NA	36.8 ± 29.8	NA	Secondary	ICD	All-cause mortality
Coleman 2008	Prospective study	USA	Hartford hospital	1997–2007	1204	Non statin 64.5 ± 13.3; stain 67.5 ± 10.8	Non statin 76.2 stain 80.7	Non statin 22.9 ± 9.1; stain 24.4 ± 8.3	NA	31.1 ± 30.7	HF	Primary or secondary	ICD	All-cause mortality
Cygankiewicz 2009	Prospective study	USA	Multicenter Automatic Defibrillator Implantation Trial II (MADIT II)	1997–2001	655	64.0 ± 10.0	84.0	28.0 ± 5.0	> 120 (40%)	63.0	MI and LVEF < 30%	Primary	ICD	All-cause mortality
Denollet 2012	Prospective study	Netherlands	Two Dutch referral hospitals	2003–2009	589	62.6 ± 10.1	81.0	≤ 35.0 (83%)	NA	38.4 (9.6–78.0)^a^	Distressed (type D)	Primary or secondary	ICD	All-cause mortality/cardiac mortality
Desai 2009	Prospective study	USA	NA	NA	209	Non statin 72.0 ± 10.0; stain 72.0 ± 11.0	79.9	Non statin 29.0 ± 7.0; stain 27.0 ± 7.0	NA	Non statin 35.0 ± 20.0; stain 32.0 ± 19.0	HF	NA	ICD/CRT-D	Appropriate shock
Echouffo 2016	Retrospective study	USA	NCDR-ICD Registry (CRT-D) + Centers for Medicare & Medicaid (ICD)	2006–2009	Non-diabetics: 11,345; diabetics: 7083	Non-diabetics: 75.4 ± 6.2; diabetics: 74.0 ± 5.8	Non-diabetics: 66.4; diabetics: 68.9	Non-diabetics: 24.2 ± 6.3; diabetics: 24.4 ± 6.2	≥ 120.0	36.0	HF	Primary	CRT-D	All-cause mortality
Eckart 2006	Retrospective study	USA	Military Health System Data Repository (MDR)	2000–2004	741	64.0 ± 14.0	80.8	NA	NA	24.0 ± 20.4	Renal insufficiency	Primary or secondary	ICD	All-cause mortality
Exner 2001	Retrospective study	Canada	Antiarrhythmics versus Implantable Defibrillators (AVID) Trial	1993–1997	457	Survived electrical storm:67.0 ± 11.0; survived other VT/VF episode: 64.0 ± 10.0; remaining patients: 65.0 ± 11.0	Survived electrical storm: 73.0; survived other VT/VF episode: 81.0; remaining patients: 76.0	Survived electrical storm: 29.0 ± 10.0; survived other VT/VF episode:30.0 ± 13.0; remaining patients: 35.0 ± 14.0	NA	31.0 ± 13.0	HF	Secondary	ICD	All-cause mortality
Fumagalli 2014	Prospective study	Italy	117 Italian cardiology centers	2004–2011	6311	NA	82.0	29.0 ± 9.0	NA	27.0 (14.0–44.0)^a^	HF	NA	ICD/CRT-D	All-cause mortality
Hager 2010	Retrospective study	USA	Two centers in USA	2000–2006	958	67.0	NA	< 40.0	NA	36.0	HF with CKD	Primary	ICD	All-cause mortality
Hess 2014	Retrospective study	USA	National Cardiovascular Data Registry’s (NCDR) ICD Registry	2006–2007	47,282	67.0 (57.0–75.0)^a^	74.8	24.9 ± 6.1	< 120 (69.2%); 120–140 (13.5%); > 140 (17.3%)	34.8 (28.8–39.6)^a^	MI + HF (LVEF < 30%) + congestive HF (LVEF < 35%)	Primary	ICD	All-cause mortality
Ho 2005	Retrospective study	USA	Loma Linda University Medical Center (LLUMC)	NA	360	62.0 ± 13.0	80.0	33.0 ± 17.0	NA	52.8 ± 44.4	Compromised left ventricular function	NA	ICD	All-cause mortality
Jahangir 2017	Retrospective study	USA	Their tertiary care center	2010–2011	904	66.7 ± 13.0	69.0	24.7 ± 7.0	NA	31.2 ± 1.2^b^	HF	Primary or secondary	ICD	All-cause mortality
Junttila 2020	Retrospective study	European	European Comparative Effectiveness Research to Assess the Use of Primary Prophylactic Implantable Cardioverter Defibrillators (EU-CERT-ICD) project	2002–2014	Non-diabetics: 2540; Diabetics: 995	Non-diabetics: 62.9 ± 11.7; diabetics: 65.7 ± 9.4	Non-diabetics: 81.5; diabetics: 83.9	Non-diabetics: 25.3 ± 6.1; diabetics: 25.7 ± 6.0	NA	38.4 ± 27.6	HF	Primary	ICD/CRT-D	All-cause mortality/appropriate shock
Lee 2007	Retrospective study	Canada	Canadian Institute for Health Information (CIHI)	1997–2003	2467	62.5 ± 13.4	78.8	NA	NA	4551 (person-years)	NA	NA	ICD	All-cause mortality
Lee 2015	Prospective study	Canada	Ontario ICD Database	2007–2011	3445	66.0 (58.0–73.0)^a^	79.7	< 35.0	126.0 (104.0–158.0)^a^	2.0 (1.5–2.0)^a^	HF	Primary	ICD/CRT-D	All-cause mortality
Morani 2013	Prospective study	Italy	Contak Italian Registry	2004–2007	266	67.0 ± 9.0	85.0	27.0 ± 5.0	165.0 ± 32.0	55.0 (41.0–64.0)^a^	HF	Primary or secondary	CRT-D	All-cause mortality
Morani 2018	Retrospective study	Italy	Eleven cardiology Italian centers	NA	821	67.0 ± 11.0	80.4	32.3 ± 11.2	NA	44.3 ± 26.5	NA	Primary or secondary	ICD/CRT-D	All-cause mortality
Perkiomaki 2015	Prospective study	USA	The Multicenter Automatic Defibrillator Implantation TrialCardiac Resynchronization Therapy (MADIT-CRT)	NA	1798	Cardiac death: 65.9 ± 10.9; non-cardiac death: 69.1 ± 9.7; alive: 64.1 ± 10.7	Cardiac death: 89.0; non-cardiac death: 82.0; alive: 74.0	Cardiac death: 22.0 ± 5.4; non-cardiac death: 23.9 ± 4.7; alive: 23.9 ± 5.2	Cardiac death: 156.2 ± 21.7; non-cardiac death: 157.9 ± 18.1; alive: 158.3 ± 19.7	48.0	Ischaemic cardiomyopathy (NYHA I-II) or nonischaemic cardiomyopathy (NYHA II) with LVEF < 30, QRS > 130	Primary or secondary	CRT-D + ICD	Cardiac mortality
Rogstad 2018	Retrospective study	USA	Medicare Advantage	2014–2015	8450	70.9 ± 8.92	72.0	NA	NA	12.0	NA	NA	ICD	All-cause mortality
Rorth 2019	Retrospective study	Danish	Danish Study to Assess the Efficacy of ICDs in Patients with Nonischaemic Systolic Heart Failure on Mortality (DANISH) trial	2008–2014	Non-diabetics: 905; diabetics: 211	Non-diabetics: 62.0 ± 10.0; diabetics: 63.0 ± 9.0	Non-diabetics:72.0; diabetics:75.0	Non-diabetics:24.2 ± 6.2; diabetics:23.4 ± 6.3	NA	68.0 (49.0–85.0)	Non-ischaemic systolic HF	Primary	ICD	All-cause mortality/cardiac mortality/appropriate therapy/inappropriate therapy
Ruwald 2013	Retrospective study	USA	Multicenter Automatic Defibrillator Implantation Trial—Reduce Inappropriate Therapy (MADIT-RIT)	2009–2011	Non-diabetics: 998; diabetics: 485	Non-diabetics: 63.0 ± 12.0; diabetics: 64.0 ± 11.0	Non-diabetics: 71.0; diabetics: 71.0	≤ 25.0: non-diabetics (50%); diabetics (46%)	NA	17.4	NA	Primary	ICD + CRT-D	Appropriate therapy/inappropriate therapy/appropriate shock/inappropriate shock/appropriate ATP/inappropriate ATP
Ruwald 2016	Retrospective study	Danish	Danish nationwide clinical registers	2007–2012	Primary: 1873; secondary: 2461	Primary: 62.2 ± 12.2; secondary: 62.3 ± 13.2	Primary: 81.0; secondary: 79.0	Primary: 29.4 ± 12.4; secondary: 40.4 ± 14.5	Primary: 103.4 ± 23.7; secondary: 102.2 ± 28.8	30.2 ± 19.8	NA	Primary or secondary	ICD	All-cause mortality/appropriate therapy
Santangelo 2020	Retrospective study	Italy	San Paolo Hospital	NA	193	66.3 ± 10.9	81.3	28.2 ± 5.2	NA	48.0 (22.8–76.6)^a^	Chronic HF and reduced LVEF	Primary	ICD/CRT-D	All-cause mortality
Seegers 2016	Retrospective study	Germany	University Medical Center Gottingen	1998–2010	1151	Male: 65.0 ± 12.0; female: 62.0 ± 15.0	81.2	Male:29.0 ± 11.0; female: 34.0 ± 13.0	Male: 123.0 ± 32.0; female: 112.0 ± 30.0	58.8 ± 32.4	HF	Primary or secondary	ICD/CRT-D	All-cause mortality/appropriate shock
Sjöblom 2016	Retrospective study	Sweden	Swedish Pacemaker Registry	2006–2011	789	65.0 ± 11.0	83.0	25.0 ± 10.0	134.0 ± 54.0	39.0 ± 18.0	Congestive HF	Primary	ICD/CRT-D	All-cause mortality
Stein 2009	Prospective study	USA	Synergistic Effects of Risk Factors for Sudden Cardiac Death (SERF) Study	2001–2004	1655	66.8 ± 11.7	82.0	31.7 ± 12.4	NA	12.5 (median)	NA	Primary or secondary	ICD	All-cause mortality
Steiner 2016	Prospectively study	Israeli	Israeli ICD Database	2010–2011	Non-diabetics: 1346; diabetics: 764	Non-diabetics: 62.2 ± 14.0; diabetics: 66.3 ± 9.4	Non-diabetics: 82.0; diabetics: 85.0	Non-diabetics: 30.5 ± 11.6; diabetics: 28.0 ± 8.3	Non-diabetics: 115.8 ± 29.8; diabetics: 124.6 ± 30.9	21.0 ± 10.2	HF	Primary or secondary	ICD/CRT-D	All-cause mortality/appropriate therapy/inappropriate therapy/appropriate shock/inappropriate shock/appropriate ATP/inappropriate ATP
Vandenberk 2016	Retrospective study	Belgium	University Hospitals of Leuven	1996–2014	727	62.5 ± 11.7	84.9	32.4 ± 12.4	131.0 ± 34.0	62.4 ± 49.2	Ischemic and dilated cardiomyopathy	Primary or secondary	ICD/CRT-D	All-cause mortality
Wasiak 2020	Retrospective study	Poland	Contemporary Modalities in Treatment of Heart Failure (COMMIT-HF)	2009–2013	Ischemic: 705; nonischemic: 368	Ischemic: 64.0 ± 10.2; nonischemic: 52.8 ± 12.9	Ischemic: 85.6; nonischemic: 74.0	Ischemic: 26.0 ± 5.7; nonischemic: 24.0 ± 5.6	NA	60.5	Systolic HF	Primary	ICD/CRT-D	All-cause mortality
Wilson 2017	Retrospective study	UK	Multicenter in Southampton and Bristol Heart Institute	2006–2014	424	> 60.0	86.3	60.0–69.9 years: 31.7 ± 15.2; 70.0–79.9 years: 26.2 ± 10.3; > 80.0 years: 31.9 ± 11.4	NA	32.6	HF	Primary	ICD/CRT-D	All-cause mortality
Winkler 2019	Retrospective study	Poland	Military Institute of Medicine in Warsaw	2011–2017	457	66.0 ± 11.0	80.6	29.0 (25.0–33.0)^a^	NA	31.0 (17.0–52.0)	HF	Primary or secondary	ICD/CRT-D	All-cause mortality/appropriate therapy
Zhang 2014	Prospective study	USA	Prospective Observational Study of Implantable Cardioverter-Defibrillators (PROSE-ICD)	NA	1189	60.6 ± 12.7	72.9	22.3 ± 7.4	118.7 ± 30.7	12.0	HF	Primary	ICD	All-cause mortality

*ICD* implantable cardioverter-defibrillator, *CRT-D* cardiac resynchronization therapy defibrillators, *HF* heart failure, *LVEF* left ventricular ejection fraction, *CKD* chronic kidney disease, *MI* myocardial infarction, *NYHA* New York Heart Association, *ATP* antitachycardia pacing, *NA* not available

^a^Medians with interquartile range

^b^Mean ± SEM

**Table 2:** NOS items scores

Study	Selection	Comparability	Outcome	Scores
Bilchick 2012	3	2	3	8
Borleffs 2009	4	2	3	9
Briongos 2019	4	1	3	8
Chao 2014	3	1	3	7
Coleman 2008	3	2	3	8
Cygankiewicz 2009	3	2	3	8
Denollet 2012	3	1	2	6
Desai 2009	4	1	3	8
Echouffo 2016	3	2	3	8
Eckart 2006	3	1	2	7
Exner 2001	3	2	3	8
Fumagalli 2014	3	1	3	7
Hager 2010	3	1	3	7
Hess 2014	4	1	3	8
Ho 2005	4	1	2	7
Jahangir 2017	3	1	3	7
Junttila 2020	3	1	3	7
Lee 2007	3	2	3	8
Lee.D 2015	4	1	3	8
Morani 2013	4	2	3	8
Morani 2018	3	1	3	7
Perkiomaki 2015	3	2	3	8
Rogstad 2018	3	2	3	8
Rorth 2019	4	2	3	9
Ruwald 2013	3	2	3	8
Ruwald 2016	3	1	3	7
Santangelo 2020	3	1	3	7
Seegers 2016	4	1	3	8
Sjöblom 2016	3	1	3	7
Stein 2009	4	1	2	7
Steiner 2016	3	1	3	7
Vandenberk 2016	3	2	3	8
Wasiak 2020	3	1	3	7
Wilson 2017	3	1	3	7
Winkler 2019	3	1	3	7
Zhang 2014	3	2	3	8

Average score: 7.55

### Increased mortality in ICD recipients with diabetes

**Fig. 2:**
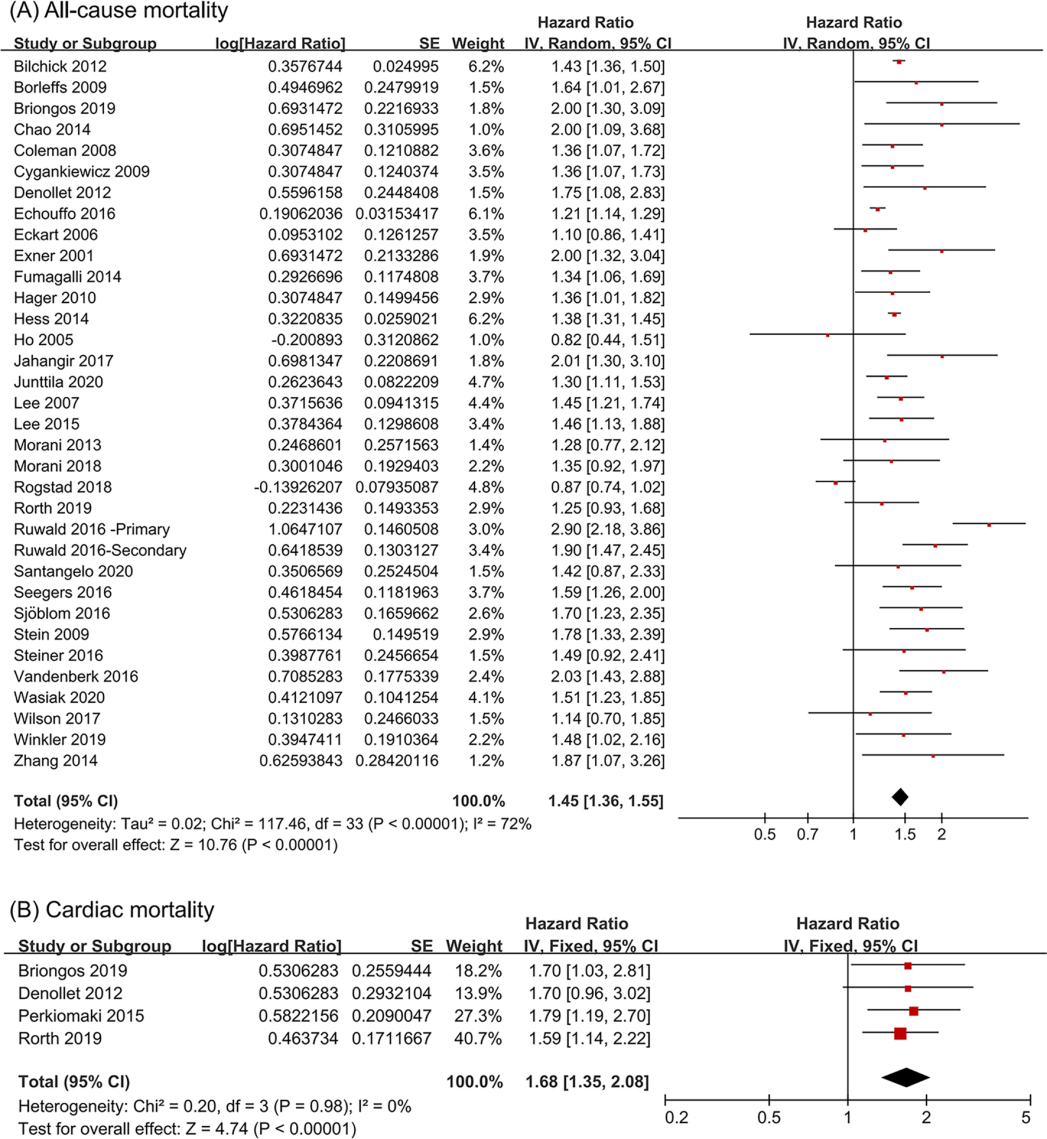
The influence of diabetes on all-cause mortality (**A**) and cardiac mortality (**B**) in ICD recipients compared with non-diabetes. *ICD* implantable cardioverter-defibrillator

In the included studies, 33 studies of 159,290 ICD recipients reported data for the association between diabetes and risk of all-cause mortality. A random effects model was used due to the existence of heterogeneity (I^2^ = 72%, P = 0.001), and the results showed that diabetes was associated with an increased risk of all-cause mortality in ICD recipients (HR = 1.45, 95% CI 1.36–1.55) (Fig. 2A). Data in 4 studies [10, 14, 29, 31] were available for cardiac mortality. The pooled data found an increased risk of cardiac mortality in ICD recipients with diabetes (HR = 1.68, 95% CI 1.35–2.08, I^2^ = 0%), shown in Fig. 2B. For the all-cause mortality outcome, funnel plots showed no significant publication bias (Additional file 1: Fig. S1). Furthermore, Begg and Egger tests also suggested no publication bias (all P > 0.1). Sensitivity analysis confirmed that the results did not change after removing individual studies (Additional file 1: Fig. S2).

### Subgroup analysis of prevention types

**Fig. 3:**
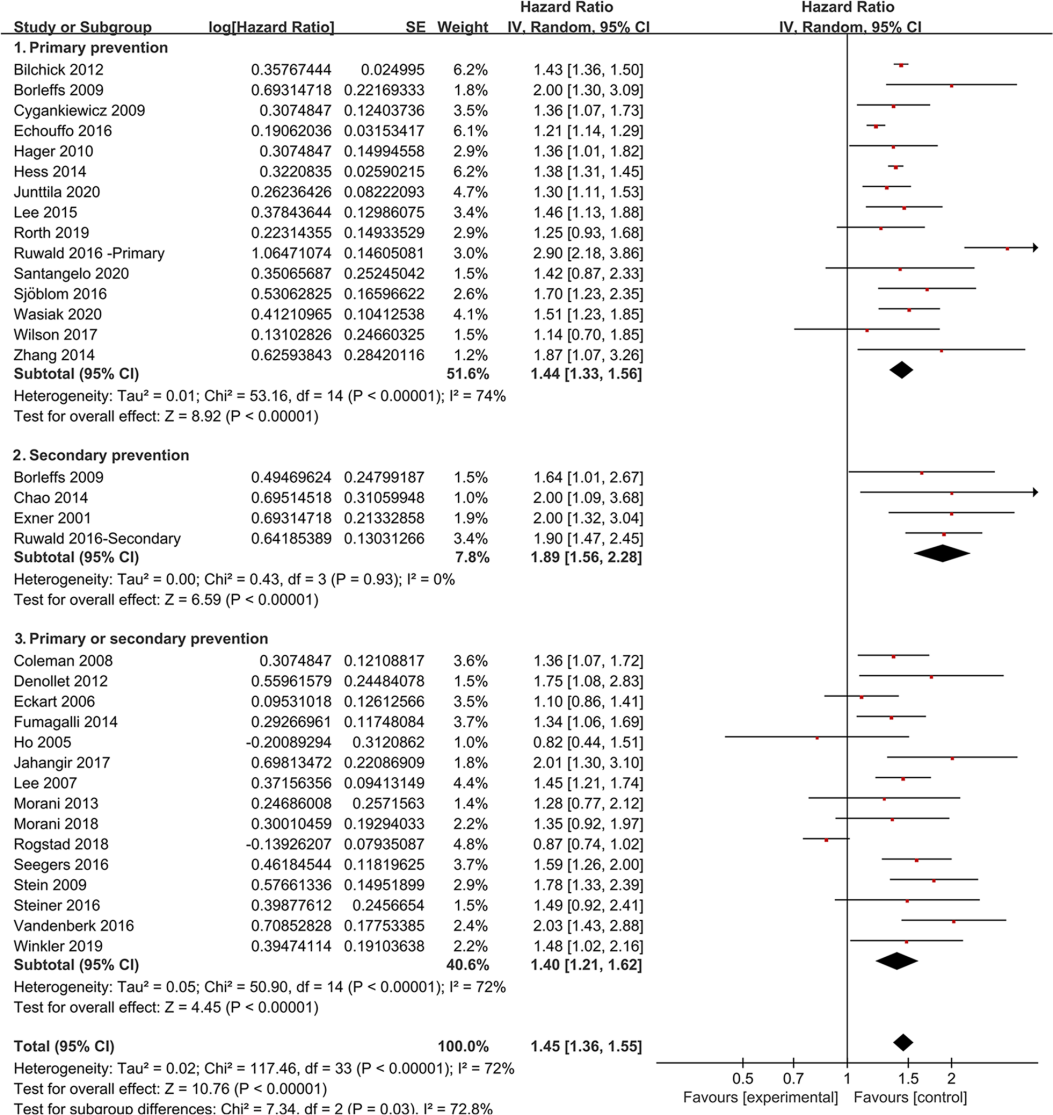
Subgroup analysis of the increased all-cause mortality caused by diabetes in ICD recipients, stratified according to primary prevention, secondary prevention and primary or secondary prevention

We performed a subgroup analysis of prevention type by separating the ICD recipients into 3 groups: ICD recipients with primary prevention, with secondary prevention and with primary or secondary prevention. Figure 3 shows that diabetes was associated with an increased risk of all-cause mortality in all 3 groups. The increase of all-cause mortality varied between the above groups (for subgroup analysis, P = 0.03), and that secondary prevention patients with diabetes may suffer a higher risk of all-cause mortality (HR = 1.89, 95% CI 1.56–2.28).

### No significant effect on ICD therapy, shock and appropriate ATP, but a decreased risk of inappropriate ATP

**Fig. 4:**
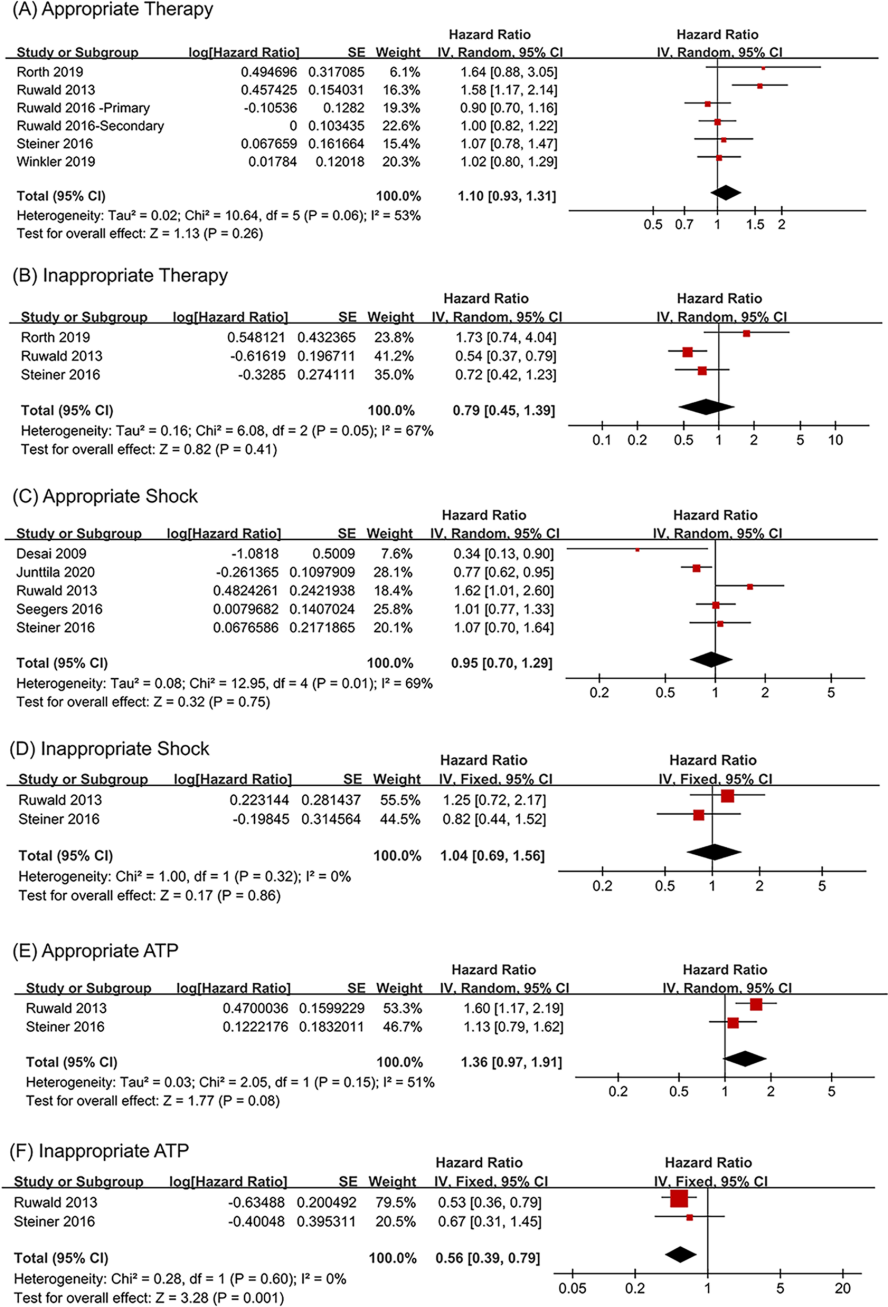
The influence of diabetes on appropriate therapy (**A**), inappropriate therapy (**B**), appropriate shock (**C**), inappropriate shock (**D**), appropriate ATP (**E**) and inappropriate ATP (**F**) in ICD recipients compared with non-diabetes. *ICD* implantable cardioverter-defibrillator *ATP* anti-tachycardia pacing

In the 36 included articles, 5 studies [31–34, 39] reported appropriate therapy, 3 studies [31, 33, 39] reported inappropriate therapy, 5 studies [15, 24, 33, 36, 39] reported appropriate shock, 2 studies [33, 39] reported inappropriate shock, ATP and inappropriate ATP. Forest plots showed that diabetes had nonsignificant relationship with the risk of appropriate therapy (HR = 1.10, 95% CI 0.93–1.31, I^2^ = 53%) (Fig. 4A), inappropriate therapy (HR = 0.79, 95% CI 0.45—1.39, I^2^ = 67%) (Fig. 4B), appropriate shock (HR = 0.95, 95% CI 0.70–1.29, I^2^ = 69%) (Fig. 4C) and inappropriate shock (HR = 1.04, 95% CI 0.69–1.56, I^2^ = 0%) (Fig. 4D) in ICD recipients. Meanwhile, no statistically significant difference was found between diabetes and the risk of ATP (HR = 1.36, 95% CI 0.97–1.91, I^2^ = 51%) (Fig. 4E) in ICD recipients. However, Fig. 4F shows that diabetes was associated with a decreased risk of inappropriate ATP (HR = 0.56, 95% CI 0.39–0.79, I^2^ = 0%).

## Discussion

The present study systematically and comprehensively reviewed the current available literature, including 36 publications with 162,780 ICD recipients, to assess the potential influence of diabetes on the mortality and risk of ICD therapy. Not as we expected, the meta-analysis indicated that in ICD recipients, diabetes was associated with an increased risk of both all-cause mortality and cardiac mortality, and secondary prevention patients with diabetes may suffer a higher risk of all-cause mortality. Another important discovery was that there were no nonsignificant differences in the proportion of ICD therapies (appropriate therapy, inappropriate therapy, appropriate shock, inappropriate shock and appropriate ATP) between diabetes patients and non-diabetes patients. However, diabetes was associated with a reduced risk of inappropriate ATP. To the best of our knowledge, this study is the first systematic review and meta-analysis to comprehensively assess the cumulative evidence of diabetes associated with mortality and the risk of ICD therapy in ICD recipients. Although there were no randomized controlled trials due to the particularity of the study design, according to the quality evaluation of the NOS, all of the included studies were of high quality. Sensitivity analysis also showed that the results were not affected by any individual studies. The above factors show the robustness of the results.

There is a high proportion of diabetes in HF patients, especially in hospitalized HF patients, and diabetes has been found to be an independent predictor of SCD in HF patients [3, 4]. On the other hand, ICD is an effective method of SCD prevention in patients with HF [6]. Based on the above theory, it can be deduced that diabetes ICD recipients with HF should receive more survival benefits than nondiabetic recipients. However, our pooled results showed that in ICD recipients, diabetes also significantly increased the risk of all-cause mortality and cardiac mortality, especially for patients with ICD implantation for secondary prevention. This result indicates that even with ICD implantation, diabetic patients still have a higher mortality than nondiabetic patients of all-cause or the cardiac mortality, which is consistent with other studies [8, 38, 39]. How to explain the increased mortality of diabetic ICD recipients is a key question. Our following work regarding whether diabetic patients have the higher risk of ICD therapies is very important to address this question, because both inappropriate and appropriate ICD therapies are associated with an increased risk of subsequent death [44–46].

ICD therapies mainly include shock and ATP. Several previous studies showed different results regarding whether diabetes increases the risk of ICD therapies. Steiner et al. showed that diabetes was not associated with an increased risk of appropriate or inappropriate ICD therapies [31, 32, 39]. However, Ruwald et al. found that patients with diabetes had a 58% increased risk of appropriate therapy and a 46% decreased risk of inappropriate therapy [33] For ICD shock and ATP, the conclusions are also not consistent [15, 24, 33, 39]. Our cumulative meta-analysis showed that diabetes ICD recipients do not have a higher risk of ICD therapies, including appropriate therapy, inappropriate therapy, appropriate shock, inappropriate shock and appropriate ATP, than nondiabetic ICD recipients. This means that the higher mortality in diabetic ICD recipients is not caused by ventricular arrhythmias or ICD therapies. Therefore, a possible reason for the increased mortality in diabetes recipients may be the comorbidities related to diabetes, independent of the effects of ICD therapy [24]. Our study found that diabetes was associated with a reduced risk of inappropriate ATP. The underlying mechanism for this phenomenon is not clear, and the possible reasons are that diabetic patients are less likely to experience exercise-induced sinus tachycardia due to reduced activity, and their cardiovascular reflexes are reduced due to autonomic nervous dysfunction and neuropathy [33].

Our results show that diabetes is significantly associated with an increased risk of mortality in ICD recipients. On the other hand, diabetes has no effect on the risk of ICD therapies. This suggests that the increased risk of mortality caused by diabetes in ICD recipients may be due to adverse pathophysiological changes and related complications caused by diabetes itself rather than arrhythmias.

Our results showed that the all-cause mortality of secondary prevention patients with diabetes was higher than diabetic primary prevention patients. A study suggested that secondary prevention patients have a higher risk of death than primary prevention patients [47], which is consistent with our finding. The results indicated that secondary prevention patients may have a vulnerable myocardium resulting from more risk factors, therefore, the vulnerable myocardium may be more likely to be damaged by diabetic complications, resulting in a higher risk of mortality. In addition, the survival benefits of ICD treatment for diabetes recipients are limited. ICD is effective in treating ventricular tachyarrhythmias; however, HF patients with diabetes may be at increased risk of mortality through mechanisms other than arrhythmias that can be treated by ICD. Our results also suggest that for these diabetes ICD recipients, more aggressive treatment should be applied to treat the adverse pathophysiological changes and complications caused by diabetes, rather than just focusing on the treatment of arrhythmias. For example, many anti-diabetic medications have been shown to improve the prognosis of diabetic patients with HF. For example, dapagliflozin, a sodium–glucose cotransporter 2 inhibitor, can significantly reduce cardiac and all-cause mortality in diabetic patients with HF [48]. Real-world studies have shown that metformin also significantly reduces mortality in diabetic patients with HF [49].

Our research has several advantages. First, to the best of our knowledge, this is the first systematic review and meta-analysis to comprehensively assess the cumulative evidence of diabetes associated with mortality and the risk of ICD therapy in ICD recipients. Second, we strictly followed the PRISMA guidelines to carry out this study. Third, all of the included studies were of high quality, and sensitivity analysis also showed the robustness of the results. Finally, such a large sample (36 studies containing 162,780 patients) can ensure the reliability of the study results. However, several limitations should be considered. First, due to the particularity of the study design, no randomized controlled trials were included. Second, there was relatively high heterogeneity among the included articles, such as in the outcomes of all-cause mortality, appropriate and inappropriate therapy, appropriate shock and ATP, which may mainly due to the individual characteristics of each included studies. Hence, we tried several ways to reduce the impact of heterogeneity on the results, including using random effects models, performing sensitivity analysis and subgroup analysis. Third, although most of the included studies adjusted for a range of confounding variables, we could not rule out an effect of residual confounding variables on the results, which may also account for the heterogeneity existence in the outcomes above.

## Conclusions

In summary, our study shows that diabetes is associated with an increased risk of mortality in ICD recipients, especially in the secondary prevention patients, but diabetes has no significant effect on the risks of ICD therapies. These results indicate that the increased mortality of ICD recipients with diabetes may not be caused by arrhythmias. The survival benefits of ICD treatment for diabetic ICD recipients are limited, and more aggressive treatment should be sought to reduce mortality.

## Supplementary Information

**Additional file 1: Figure S1.** Funnel plot of the outcome (all-cause mortality). **Figure S2.** Sensitivity of the outcome (all-cause mortality).

## Data Availability

None.

## Abbreviations

ICD: Implantable cardioverter-defibrillator
HRs: Hazard ratios
CIs: Confidence intervals
HF: Heart failure
SCD: Sudden cardiac death
ATP: Anti-tachycardia pacing
LVEF: Left ventricular ejection fractions
NOS: Newcastle–Ottawa Scale
PRISMA: Preferred Reporting Items for Systematic Reviews and Meta-Analyses
NYHA: New York Heart Association
RevMan: Review Manager
