# Supplementary material for: Influence of diabetes on mortality and ICD therapies in ICD recipients: a systematic review and meta-analysis of 162,780 patients
Source: Cardiovasc Diabetol. 2022 Jul 29;21:143. doi: 10.1186/s12933-022-01580-y (PMC9338523; doi:10.1186/s12933-022-01580-y)

**Additional Files**

**Additional file 1: Figure S1.** Funnel plot of the outcome (all-cause mortality).


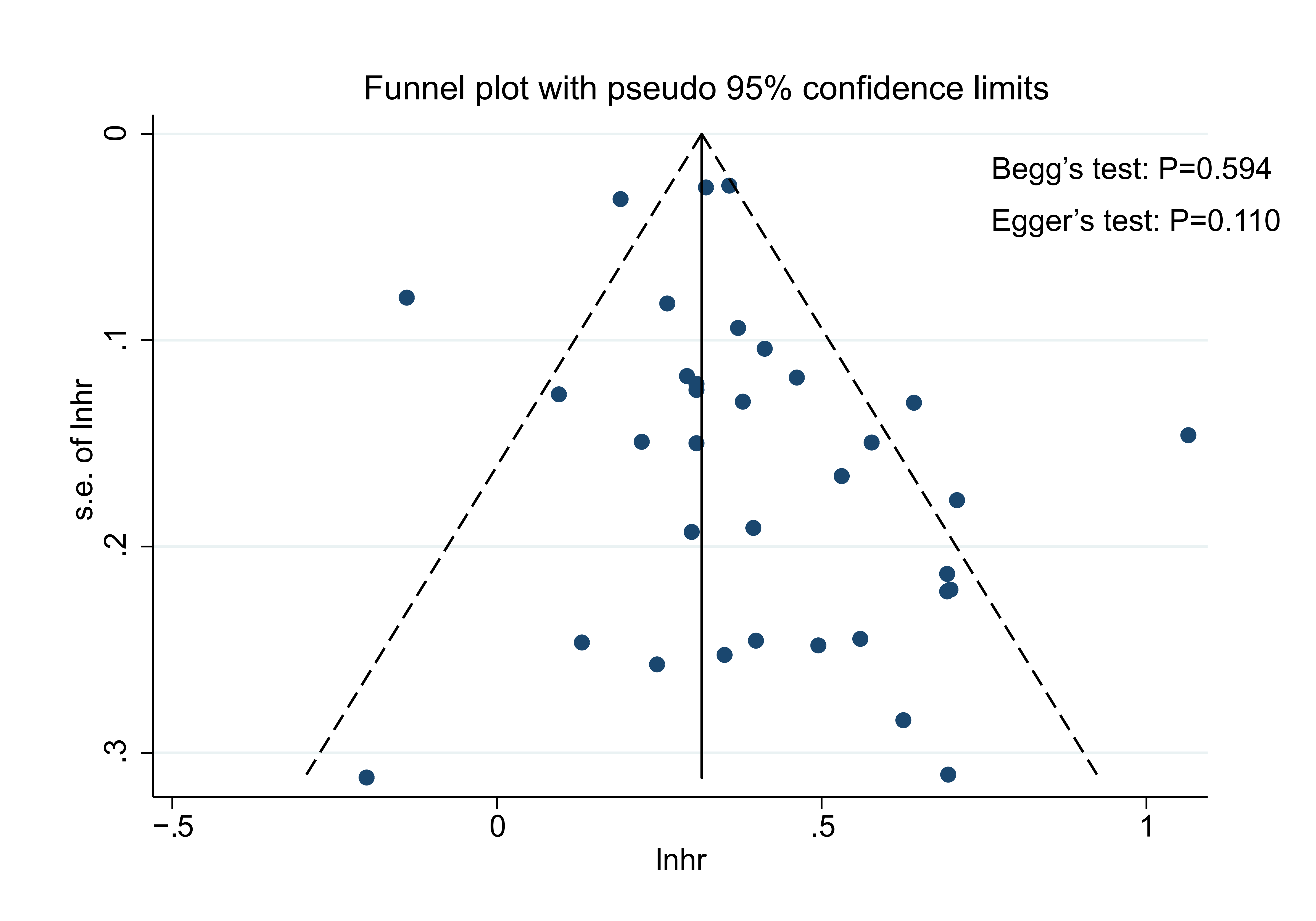


**Additional file 1: Figure S2.** Sensitivity of the outcome (all-cause mortality).


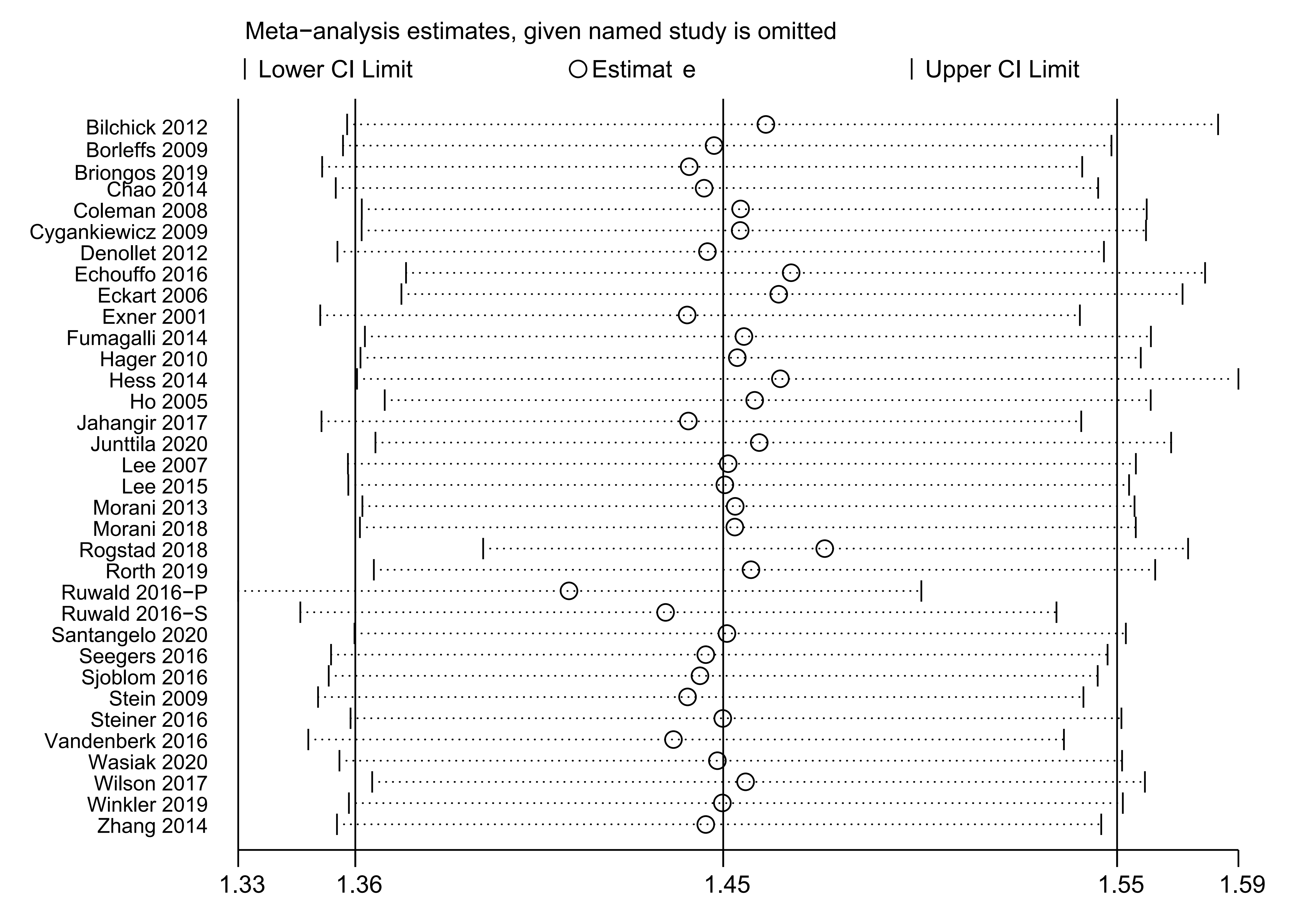

Supplement: Supplementary file 1 — Additional file 1: Figure S1. Funnel plot of the outcome (all-cause mortality). Figure S2. Sensitivity of the outcome (all-cause mortality). [file 12933_2022_1580_MOESM1_ESM.docx]
